# Lipid A modification of colistin-resistant *Klebsiella pneumoniae* does not alter innate immune response in a mouse model of pneumonia

**DOI:** 10.1128/iai.00016-24

**Published:** 2024-05-21

**Authors:** Gitanjali Bhushan, Victor Castano, Tania Wong Fok Lung, Courtney Chandler, Thomas H. McConville, Robert K. Ernst, Alice S. Prince, Danielle Ahn

**Affiliations:** 1 Department of Pediatrics, Columbia University Irving Medical Center, New York, New York, USA; 2 Department of Microbial Pathogenesis, University of Maryland, School of Dentistry, Baltimore, Maryland, USA; 3 Department of Medicine, Columbia University Irving Medical Center, New York, New York, USA; Stanford University School of Medicine, Stanford, California, USA

**Keywords:** *Klebsiella pneumoniae*, pulmonary infection, polymyxin, colistin, *mcr-1*, lipid A, host immune response, antimicrobial resistance, carbapenem resistance, colistin resistance

## Abstract

Polymyxin resistance in carbapenem-resistant *Klebsiella pneumoniae* bacteria is associated with high morbidity and mortality in vulnerable populations throughout the world. Ineffective antimicrobial activity by these last resort therapeutics can occur by transfer of *mcr-1*, a plasmid-mediated resistance gene, causing modification of the lipid A portion of lipopolysaccharide (LPS) and disruption of the interactions between polymyxins and lipid A. Whether this modification alters the innate host immune response or carries a high fitness cost in the bacteria is not well established. To investigate this, we studied infection with *K. pneumoniae* (KP) ATCC 13883 harboring either the *mcr-1* plasmid (p*mcr-1*) or the vector control (pBCSK) ATCC 13883. Bacterial fitness characteristics of *mcr-1* acquisition were evaluated. Differentiated human monocytes (THP-1s) were stimulated with KP bacterial strains or purified LPS from both parent isolates and isolates harboring *mcr-1*. Cell culture supernatants were analyzed for cytokine production. A bacterial pneumonia model in WT C57/BL6J mice was used to monitor immune cell recruitment, cytokine induction, and bacterial clearance in the bronchoalveolar lavage fluid (BALF). Isolates harboring *mcr-1* had increased colistin MIC compared to the parent isolates but did not alter bacterial fitness. Few differences in cytokines were observed with purified LPS from *mcr-1* expressing bacteria *in vitro*. However, in a mouse pneumonia model, no bacterial clearance defect was observed between p*mcr-1*-harboring KP and parent isolates. Consistently, no differences in cytokine production or immune cell recruitment in the BALF were observed, suggesting that other mechanisms outweigh the effect of these lipid A mutations in LPS.

## INTRODUCTION

Multidrug-resistant (MDR) *Klebsiella pneumoniae* (KP) are Gram-negative organisms that are difficult to treat and cause infections associated with high morbidity and mortality (1, 2). Most infections occur in hospitalized patients, with several outbreaks reported in long-term care facilities and community-onset infection in immunocompromised patients with extensive exposure to health care (3). Recently, MDR KP emerged as a significant cause of ventilator-associated pneumonia in coronavirus disease 2019 acute respiratory distress syndrome (4). The overuse of antibiotics in these virally infected patients with little regard for antibiotic stewardship had disastrous effects, leading to outbreaks of MDR pathogens throughout the world (5). With a paucity of therapeutic options, polymyxins such as colistin have become the last resort treatment, despite their high risk of neurotoxicity and nephrotoxicity (6). Consequentially, these genetically promiscuous organisms have been further selected for isolates resistant to these precious therapies, leading to the persistence of colistin-resistant KP strains globally (7).

Colistin (polymyxin E) has bactericidal activity against Gram-negative bacilli, including *Klebsiella*, *Pseudomonas*, *Acinetobacter*, and *Enterobacter* species. The target site of colistin is the bacterial outer membrane, with the outer leaflet composed mainly of lipopolysaccharide (LPS), a well-characterized pathogen-associated molecular pattern in Gram-negative bacteria. Polymyxins (including colistin or polymyxin E) are cationic, surface-active agents that disrupt the structure of cell membrane phospholipids and increase cell permeability by a detergent-like action (8). In colistin-sensitive bacteria, the outer membranes are permeabilized and allow for bacterial lysis and death (8). Colistin resistance is generally chromosomally derived; however, plasmid-mediated, transmissible resistance genes, such as mobilized colistin resistance (*mcr-1*), that encodes a phosphoethanolamine (pEtN) transferase also affect the efficacy of colistin (9, 10). This enzyme adds a pEtN moiety to the terminal phosphate portion of lipid A, the immunogenic portion of LPS, creating an unfavorable electrostatic interaction between colistin and its target LPS, thereby increasing the likelihood of bacterial survival (11). Persisting stably on a plasmid and exchanged easily between isolates, *mcr-1* has rapidly disseminated resistance to colistin in KP isolates worldwide (7).

Beyond antimicrobial resistance, modifications to LPS have the potential to alter the host inflammatory response by changing the interaction of LPS with the TLR4–MD2 complex, and therefore downstream host inflammatory signaling (12). However, it is not well established whether alterations in LPS composition due to *mcr-1* expression in KP disrupt the production of inflammatory effectors and immune cell recruitment, or carry a high fitness cost (13, 14). Previous studies have shown decreased fitness and attenuated cytokine production in a *Galleria mellonella* infection model and reduced cytokine production in human-derived THP-1 cells infected with *Escherichia coli* expressing *mcr-1* (14). Other modifications to LPS that confer colistin resistance, such as adding 4-amino-4-deoxy-l-arabinose (Ara4N), have been shown to enhance intracellular survival after phagocytosis and promote inflammatory signaling (15). In this study, we show that KP strains harboring the *mcr-1* plasmid have an increased resistance to colistin with differential production of cytokines in human monocytes *in vitro*. The change in lipid A conferred by *mcr-1*, however, does not alter immunogenicity, bacterial clearance, and immune cell recruitment in an *in vivo* model of murine bacterial pneumonia, suggesting that other mechanisms outweigh the effect of these changes in LPS.

## MATERIALS AND METHODS

### Cell lines and bacterial strains

Immortalized human monocytes (THP-1, ATCC) were grown at 37°C with 5% CO_2_ in Roswell Park Memorial Institute (Corning) with 10% heat-inactivated fetal bovine serum (Gibco) and 1% penicillin/streptomycin (Corning). Purified *E. coli* LPS (Sigma-Aldrich) and *K. pneumoniae* 13883, as well as *mcr-1* LPS, were used at 10 µg/mL concentrations. *K. pneumoniae* ATCC 43816 (KPPR1), ATCC 13883, KP35, and *P. aeruginosa* ATCC 47085 (PAO1) were grown in lysogenic broth (LB) and resuspended in phosphate buffered saline (PBS) for *in vitro* assays and *in vivo* infections. Isolates harboring pBCSK and p*mcr-1* were grown in 20 µg/mL chloramphenicol. The concentration of bacteria for each was enumerated by serial dilution and plating on LB agar. *K. pneumoniae* ATCC 13883 was obtained from ATCC, whereas 13883 + *mcr-1* and 13883 + pBCSK were generously provided by Dr. Robert K. Ernst.

### Colistin susceptibility testing

MICs were determined using broth microdilution according to CLSI guidelines. Briefly, bacterial isolates were resuspended in normal saline to 0.5 McFarland, diluted 1:100 in cation-adjusted Mueller-Hinton broth (CAMHB), and inoculated into a 96-well plate containing increasing concentrations of colistin diluted in CAMHB. Plates were incubated for 20–24 hours at 37°C. MIC determination corresponded to the well where the bacterial button was at least less than half the size of the previous well. *In vitro* colistin resistance was defined as an MIC >2 mg/L (16).

### Kinetic growth curve

Bacterial strains were grown overnight in LB media shaking at 37°C in a sterile tube. Bacteria were pelleted, resuspended in PBS at an optical density (OD) of 0.5, and incubated in a 1:100 dilution in LB media in a round-bottom 96-well plate with a final volume of 100 µL per well. Plates were incubated under agitation at 37°C overnight for kinetic growth curves. OD_600_ was measured every 30 minutes for 18 hours on a SpectraMax M2 Microplate reader (Molecular Devices, Toronto, Canada).

### Biofilm

To measure biofilm formation, bacteria were standardized to an OD_600_ of 1, inoculated 1:100 in trypticase soy media supplemented with 0.5% glucose in a 96-well plate, and grown statically overnight at 37°C. After 24 hours, OD_600_ was measured, plates were washed twice, incubated for 15 minutes with 100% methanol, washed again followed by the addition of 1% crystal violet, and then resuspended with 33% acetic acid. Plates were then measured at OD_600_. To analyze biofilm production, OD_540_/OD_600_ was calculated.

### Bacterial reactive oxygen species production

Bacterial strains were grown overnight in LB and were then subcultured 10:100 in LB, incubated, standardized to an OD_600_ of 0.5, washed, and resuspended in 1 mL PBS. Two hundred microliters of resuspended bacteria was added in quadruplicate to a V-bottom 96-well plate. MitoSOX (Thermo Fisher) was added to bacterial cells and incubated for 20 minutes, before pelleting bacteria and washing twice with PBS. Bacterial cells were added to fluorescent-activated cell sorting (FACS) tubes on ice before FACS analysis on BD FACSCanto II.

### LPS purification

Large-scale LPS preparations were isolated using a hot phenol/water extraction method after growth in LB supplemented with 1 mM MgCl_2_ at 37°C (17). Subsequently, LPS was treated with RNase A, DNase I, and proteinase K to ensure purity from contaminating nucleic acids and proteins (18). Individual LPS samples were additionally extracted to remove contaminating phospholipids (19) and toll-like receptor 2 (TLR2) contaminating proteins (20). For structural analysis, lipid A was isolated after hydrolysis in 1% sodium dodecyl sulfate (SDS) at pH 4.5, as described (21). Briefly, 500 µL of 1% SDS in 10 mM Na-acetate at pH 4.5 was added to a lyophilized sample. Samples were incubated at 100°C for 1 hour, frozen, and lyophilized. The dried pellets were resuspended in 100 µL of water and 1 mL of acidified ethanol (100 µL 4N HCl in 20 mL 95% EtOH). Samples were centrifuged at 5,000 rpm for 5 minutes. The lipid A pellet was further washed (3×) in 1 mL of 95% EtOH. The entire series of washes were repeated twice. Samples were resuspended in 500 µL of water, frozen on dry ice and lyophilized before mass spectrometric analysis.

### THP-1 cell studies

Bacteria were grown in LB and standardized to an OD_600_ of 0.5 and added to THP-1 cells at a multiplicity of infection (MOI) 10 for 6 hours. Ten micrograms per milliliter of purified *K. pneumoniae* 13883 and *mcr-1* LPS was added to THP-1s for 1 hour. Cells supernatants were removed, centrifuged, and then aliquoted for a 10-plex human and mouse discovery assay cytokine analysis by Eve Technologies (Alberta, Canada).

### Mouse studies

*In vivo* experiments were performed using 8-week-old male C57BL/6J mice (Jackson Laboratories). Mice were anesthetized with 100 mg/kg ketamine and 5 mg/kg xylazine given intraperitoneally and infected intranasally with 13883, pBCSK, p*mcr-1* (10^7^ CFU in 50 µL of PBS). Bacterial load of spleen, lung, and bronchoalveolar lavage fluid (BALF) was quantified by serial dilutions on LB agar. Animal experiments were performed in accordance with the guidelines of the IACUC at Columbia University (protocol number AABG558).

### BALF assays

BALF was obtained by passing aliquots of sterile PBS with calcium and magnesium into a cannulated trachea. Serial dilutions for bacterial enumeration were performed on the BALF prior to centrifuging. One milliliter of recovered BALF supernatant was used for cytokine array, a 10-plex mouse discovery assay from Eve Technologies (Alberta, Canada). Total cellular pellet content was used for flow cytometry (FC).

### Analysis of cell populations

To identify immune cell populations in BALF and lung homogenate, multicolor FC was conducted on a BD LSRII in the CCTI Flow Cytometry Core (NIH S10RR027050). Cells were stained with BV605-labeled anti-CD11c, AF700-labeled anti-CD45, AF594-labeled anti-CD11b, AF647-labeled anti-Siglec F, APC-Cy7-labeled anti-MHCII, phycoerythrin (PE)-Cy7-labeled anti-F4/80, BV421-labeled anti-Ly6C, PerCP-Cy5.5-labeled anti-Ly6G, Live/Dead Fixable Dead Cell Stain Kits, blue fluorescent dye (Invitrogen), and Fc block (anti-mouse CD16/32). All antibodies were purchased from Biolegend (San Diego, California, USA). Uniform dyed microspheres were added to calculate the concentration of distinct immune cell populations (Bang Laboratories). All FC data were analyzed on FlowJo (Version 10.0.8).

## RESULTS

### Acquisition of p*mcr-1* increases colistin MIC but does not change the basic characteristics of *K. pneumoniae*

**Fig 1:**
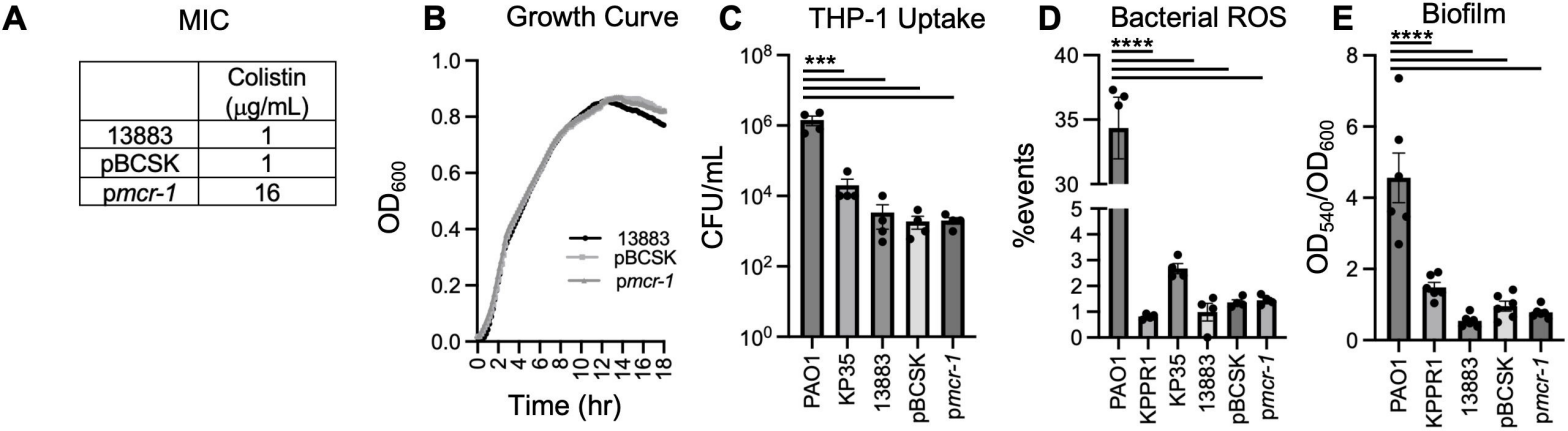
MIC and basic characteristics of WT *K. pneumoniae* and p*mcr-1.* (**A**) MIC of WT *K. pneumoniae* ATCC 13883, pBCSK, and p*mcr-1* to colistin. (**B**) Growth kinetics of ATCC 13883, pBCSK, and p*mcr-1.* (**C**) Bacterial uptake into human monocytes (THP-1) by *Pseudomonas aeruginosa* PAO1, ATCC 43816 (KPPR1), clinical isolate ST258 (KP35), ATCC 13883, pBCSK, and p*mcr-1*. (**D**) Bacterial reactive oxygen species production. (**E**) Biofilm production. Data are shown as average ± SEM from three independent experiments. Significance was determined by one-way analysis of variance (ANOVA) with multiple comparisons; ***P < 0.001 and ****P < 0.0001.

The lipid A structures of LPS produced by ATCC 13883, a laboratory reference strain, with the empty plasmid (pBCSK) and p*mcr-1* were previously characterized (11). To confirm the effect of *mcr-1* plasmid acquisition, the MIC to colistin by microdilution method of ATCC 13883 with p*mcr-1* (16 µg/mL) was compared to the empty plasmid control (pBCSK) (1 µg/mL). This difference indicates that the addition of the *mcr-1* plasmid to KP confers greater resistance (~16-fold) to colistin (Fig. 1A). Alteration in the lipid A structure of LPS did not change the basic characteristics of KP by evaluation of growth kinetics in nutrient-rich media (Fig. 1B), uptake of the bacteria into differentiated human monocyte (THP-1) cells (Fig. 1C), bacterial production of reactive oxygen species (Fig. 1D), or production of biofilm (Fig. 1E). In conclusion, based on the experiments performed here, there was no apparent fitness cost for KP acquiring the *mcr-1* plasmid.

### Few differences in cytokines produced by differentiated human monocytes are produced in response to purified *mcr-1* LPS and *K. pneumoniae* harboring the *mcr-1* plasmid

**Fig 2:**
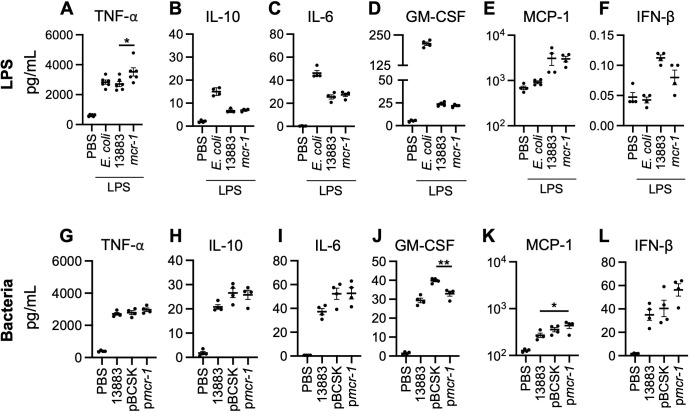
Purified *mcr-1* LPS and *K. pneumoniae* harboring the *mcr-1* plasmid in differentiated human monocytes (THP-1). Cytokine production in human monocytes (THP-1) (**A–F**) treated with 10 µg/mL purified *E. coli* and *K. pneumoniae* LPS for 6 hours. Data are shown as average ± SEM from three independent experiments. Significance was determined by one-way ANOVA with multiple comparisons; *P < 0.05. Cytokine production in human monocytes (THP-1) (**G–L**) infected with MOI 10 *K*. *pneumoniae* bacteria for 6 hours. Data are shown as average ± SEM from three independent experiments. Significance was determined by one-way ANOVA with multiple comparisons; *P < 0.05 and **P < 0.01.

Cytokine production in response to infection with Gram-negative organisms is determined by the interaction of LPS with host TLR4/MD2 receptors (22). To look specifically at how modifications in lipid A affect cytokine induction, differentiated human monocytes to macrophage phenotype (THP-1s) were treated with 10 µg/mL of *E. coli* LPS or LPS purified from KP ATCC 13883 with or without the *mcr-1* plasmid for 6 hours. Treatment of THP-1s with LPS produced from bacteria expressing *mcr-1* (*mcr-1* LPS) had enhanced tumor necrosis factor alpha (TNF-α) production in comparison to LPS from ATCC 13883 (*P* < 0.05) (Fig. 2A). No differences in interleukin 10 (IL-10), interleukin 6 (IL-6), granulocyte-macrophage colony-stimulating factor (GM-CSF), monocyte chemoattractant protein 1 (MCP-1), or interferon-beta (IFN-β) (Fig. 2B through F) levels were measured between *mcr-1* LPS treated cells in comparison to 13883 LPS.

To evaluate the effect of LPS modifications in infection with live bacteria, THP-1 cells were infected with the ATCC 13883 containing the *mcr-1* plasmid (p*mcr-1*) for 6 hours (MOI 10) and compared to infection with the parent strain with and without the empty plasmid (pBCSK). A significant decrease in GM-CSF was seen in the cell culture supernatant of THP-1s infected with p*mcr-1*-containing isolates compared to the empty vector plasmid, pBCSK (*P* < 0.01) (Fig. 2J). No differences in TNF-α, IL-10, IL-6, or IFN-β (Fig. 2G through I and L) levels were measured between groups. MCP-1, however, was enhanced in p*mcr-1*-containing bacteria in comparison to 13883 (*P* < 0.05) (Fig. 2K).

### Lipid A modifications in *K. pneumoniae* do not alter bacterial clearance, cytokine production, immune cell recruitment, or metabolomes in a mouse model of pneumonia

**Fig 3:**
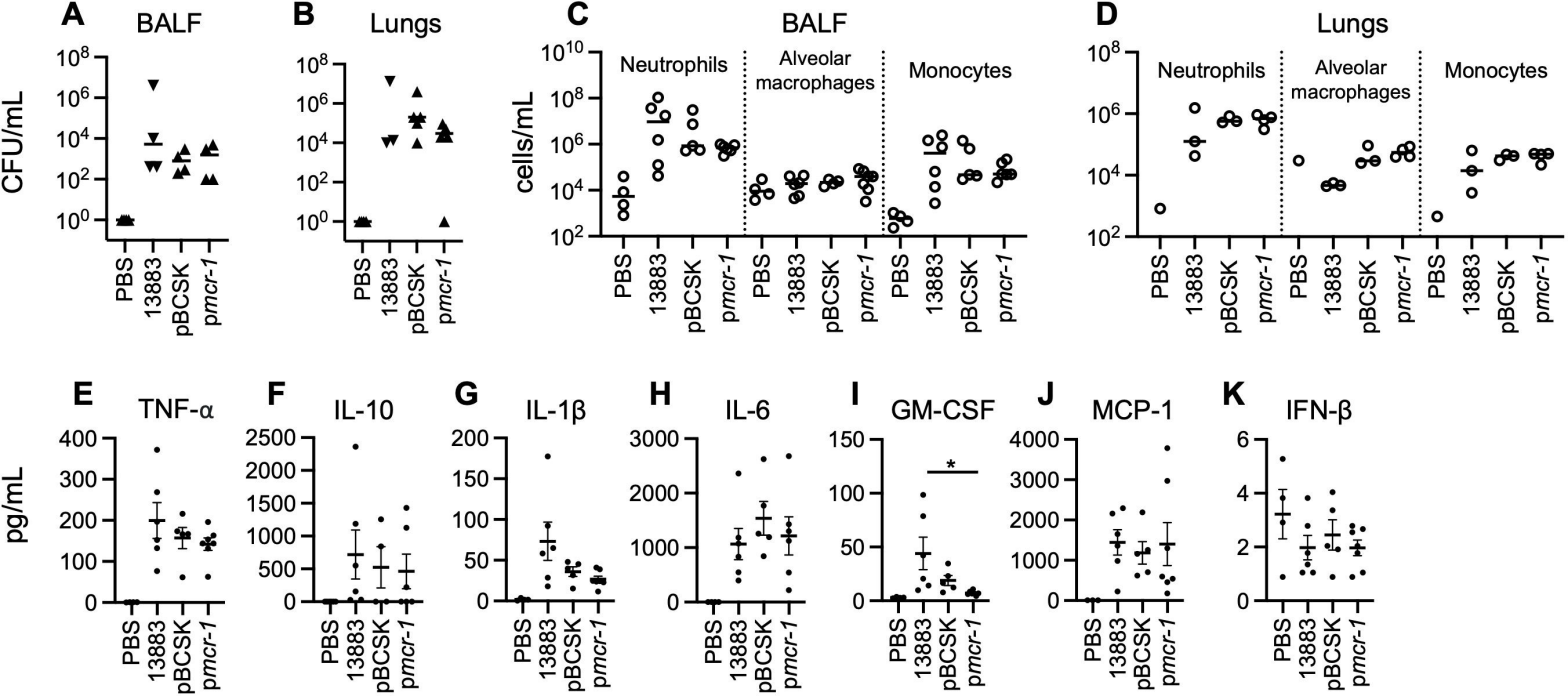
*K. pneumoniae* with modified lipid A does not alter cytokine production in BAL fluid of mice and does not modify bacterial colonization and immune cell recruitment in mouse BAL fluid and lung tissue. Bacterial burden evaluated in (**A**) BALF and (**B**) lung of C57BL/6J mice infected with 10^7^ CFU *K*. *pneumoniae*. Immune cell recruitment in (**C**) BALF neutrophils (CD45^+^CD11b^+^MHCII^lo^Ly6C^hi^Ly6G^hi^), alveolar macrophages (CD45^+^SiglecF^+^CD11b^lo-mid^), and monocytes (CD45^+^CD11b^+^MHCII^lo^Ly6C^hi^Ly6G^lo^) or (**D**) lung neutrophils (CD45^+^CD11b^+^MHCII^lo^Ly6C^hi^Ly6G^hi^), alveolar macrophages (CD45^+^SiglecF^+^CD11b^lo-mid^), monocytes (CD45^+^CD11b^+^MHCII^lo^Ly6C^hi^Ly6G^lo^) of C57BL/6J mice infected with 10^7^ CFU *K. pneumoniae*. The data shown are individual experiments representative of two independent experiments ± SEM. Significance was determined by one-way ANOVA with multiple comparisons. Cytokine production in BALF of C57BL/6J mice (**E–K**) infected with 10^7^ CFU *K*. *pneumoniae* for 48 hours. The data shown are individual experiments representative of two independent experiments ± SEM. Significance was determined by one-way ANOVA with multiple comparisons; *P < 0.05.

To determine whether changes in the lipid A structure of LPS produced by KP alter bacterial clearance, BALF and lung homogenate were collected after 48 hours of infection from C57BL/6J mice intranasally inoculated with 10^7^ CFU of the tested isolates. There was no difference in CFUs recovered from the BALF or lung homogenate between mice infected with ATCC 13883 containing p*mcr-1* or pBCSK (Fig. 3A and B). Equivalent numbers of monocytes (CD45^+^CD11b^+^MHCII^lo^Ly6C^hi^Ly6G^lo^), alveolar macrophages (CD45^+^SiglecF^+^CD11b^lo-mid^), and neutrophils (CD45^+^CD11b^+^MHCII^lo^Ly6C^hi^Ly6G^hi^) in BALF and lung homogenate of infected mice were enumerated between groups (Fig. 3C and D). There was also no difference in cytokine production of TNF-α, IL-10, IL-1β, IL-6, MCP-1, and IFN-β levels in the BALF supernatant between groups (Fig. 3E through H, J, and K). Despite no changes in granulocyte numbers as seen by neutrophils and monocytes recovered in the BALF and lung of infected mice, there was a statistically significant decrease in GM-CSF in the BALF of mice infected with p*mcr-1*-containing bacteria in comparison to 13883 (*P* < 0.05) (Fig. 3I). Finally, the airway metabolomes measured in the BALF supernatant were similar (Fig. S1).

## DISCUSSION

Several Gram-negative bacteria use lipid A modifications as an immune evasion strategy, contributing to their success as human pathogens (23–25). KP, like *E. coli* has a hexa-acylated LPS, which allows for a strong agonistic proinflammatory response upon binding with TLR4/MD2 expressed on a host immune cell. Any modifications in the structure of lipid A can directly alter its recognition by the TLR4–MD2 receptor and subsequent downstream immune signaling (22). Indeed, the removal of acyl chains and phosphate groups on lipid A by acquired enzymes, such as pEtN transferase, has been shown to lead to impaired recognition by pathogen recognition receptors or a shift in the type of cytokine response induced (26). For example, *Francisella tularensis*, lacking both phosphate groups, are weak agonists of TLR4 and have immunologically silent properties (27–29). Other Gram-negative bacteria, such as *Neisseria meningitis*, *Shigella flexneri*, and *Yersinia pestis* express reduced numbers of acyl chains on the LPS produced and poorly induce TLR4/MD2 engagement, resulting in a weak immune response and impaired bacterial clearance (30–32). Therefore, it is generally accepted that Gram-negative immunogenicity can be highly dependent on the degree of lipid A modification.

We, therefore, hypothesized that KP harboring *mcr-1* plasmid would attenuate immune responses due to the resultant negative charge that occurs from adding a pEtN group to lipid A. Few studies have demonstrated the specific effect of *mcr-1* gene expression on immunomodulation via changes in cytokine production. Yang et al. showed that adding *mcr-1* to *E. coli* reduces IL-6 and TNF production in differentiated monocytes (THP-1s) (13). Supporting this finding, Mattiuz et al. showed that *E. coli* harboring the *mcr-1* gene had significantly decreased production of TNF and IL-1β, while increased IL-10 abundance (14). Interestingly, attenuation of inflammation is not universally seen in infections with other Gram-negative organisms that have added pEtN to LPS. This modification in *Neisseria gonorrhoeae* infection decreases autophagy (33) and in *Salmonella enterica*, it enhances overall virulence (34). Alternatively, a recent study showed that *E. coli* harboring *mcr-1* had a functional reduction in LPS biosynthesis pathways suggesting that altered metabolic processes may decrease LPS production and subsequently decrease immunogenicity (35). Altogether, while some generalizations exist, the effect of *mcr-1* on bacterial immunogenicity and virulence is specific to the bacterial species and the infected compartment in the host.

The lack of difference in bacterial clearance and inflammatory signaling in the host, as determined by changes in LPS structure alone, highlights the complexity of these host-pathogen interactions. More recent reports have underscored the important role of the shared host and pathogen metabolic environment in the context of host immunity in KP infection and persistence, particularly those belonging to the highly successful clonal complex ST258 (36, 37). Indeed, if the metabolic milieu is the most important factor in determining the degree of KP pathogenesis in the lung, our finding that LPS modification alone did not alter the airway metabolome explains the lack of clearance phenotype. From a global perspective, this may explain why *mcr-1*-containing strains are less successful human pathogens than KP ST258 isolates. While treating patients infected with *mcr-1* harboring isolates is very challenging, if the host response is somewhat equivalent, then epidemiologic interventions such as antibiotic stewardship and isolation practices may be the most effective way to limit the spread of these infections.

## SUPPLEMENTAL MATERIAL

Fig. S1 iai.00016-24-s0001.tiff Metabolomics in BALF recovered from mice infected with KP harboring p*mcr-1*.

Supplemental legend iai.00016-24-s0002.pdf Legend for Fig. S1.

ASM does not own the copyrights to Supplemental Material that may be linked to, or accessed through, an article. The authors have granted ASM a non-exclusive, world-wide license to publish the Supplemental Material files. Please contact the corresponding author directly for reuse.
