# Supplementary figures and images for: Lipid A modification of colistin-resistant Klebsiella pneumoniae does not alter innate immune response in a mouse model of pneumonia
Source: Infect Immun. 2024 May 21;92(6):e00016-24. doi: 10.1128/iai.00016-24 (PMC11237409; doi:10.1128/iai.00016-24)

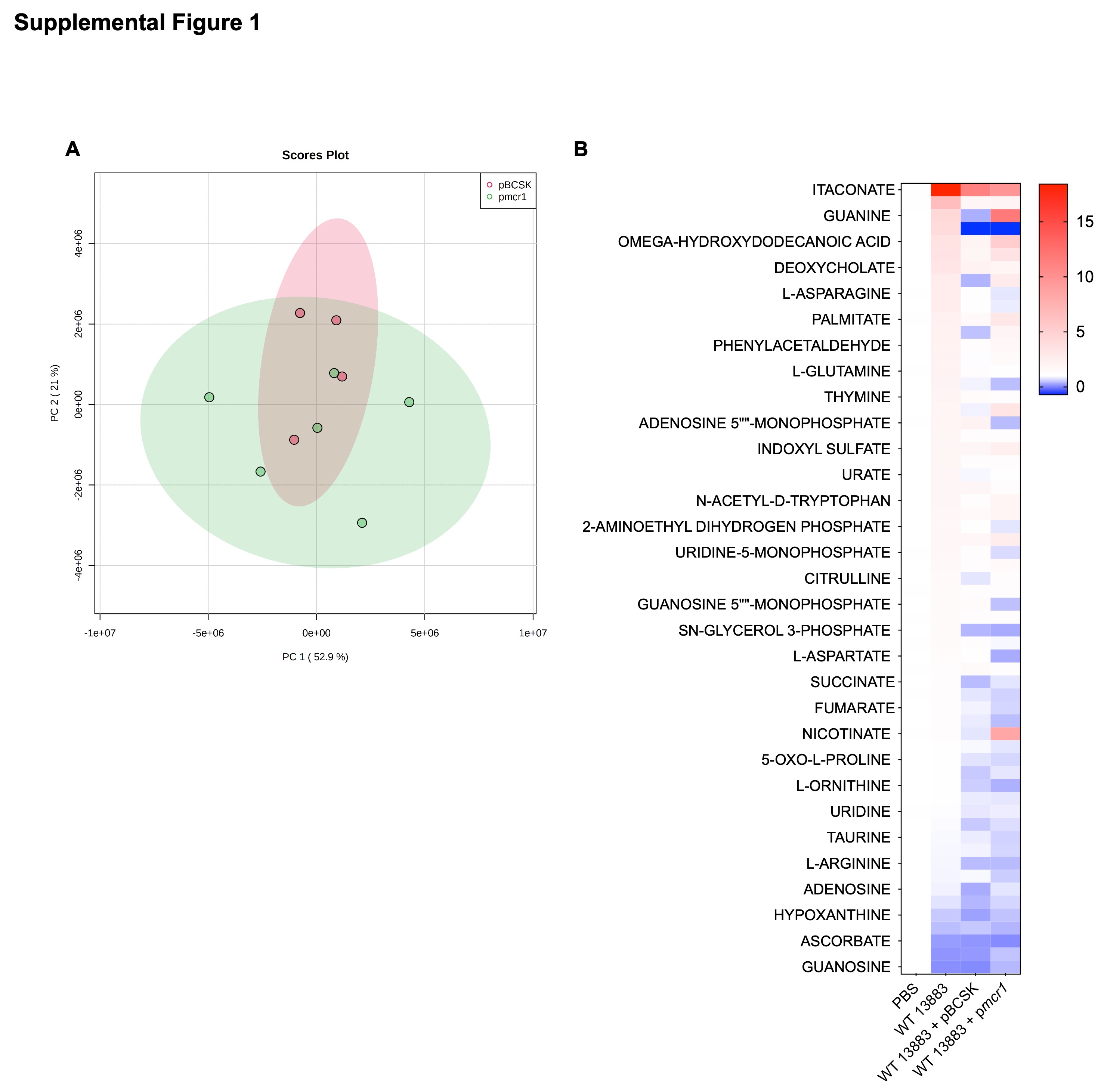

Supplement: Fig. S1 — Metabolomics in BALF recovered from mice infected with KP harboring pmcr-1. [file iai.00016-24-s0001.tiff]
