# Supplementary material for: Lipid A modification of colistin-resistant Klebsiella pneumoniae does not alter innate immune response in a mouse model of pneumonia
Source: Infect Immun. 2024 May 21;92(6):e00016-24. doi: 10.1128/iai.00016-24 (PMC11237409; doi:10.1128/iai.00016-24)
Supplement: Supplemental legend — Legend for Fig. S1. [file iai.00016-24-s0002.pdf]

Supplemental Figure 1: Airway metabolomes were not different in the presence of p*mcr-1*.

Polar metabolites from BALF supernatant from infected mice presented in Figure 3 were measured via targeted LC/MS of polar metabolites. (A) A PCA plot of the metabolites measured from the BALF of these mice (n = 5-6, each point represents 1 mouse). (B) Selected peak metabolite levels are shown via heatmap.
